# The endometrioma paradox

**DOI:** 10.5935/1518-0557.20240090

**Published:** 2025

**Authors:** João Antonio Dias Jr, Carlos Roberto Izzo, Georges Fassolas, Luiz Fernando de Oliveira Henrique, Gustavo Arantes Rosa Maciel, Sérgio Podgaec, Luciana Carvalho Delamuta

**Affiliations:** 1 Clínica Originare, Medicina Reprodutiva, São Paulo, Brazil; 2 Hospital Israelita Albert Einstein, São Paulo, Brazil

**Keywords:** endometrioma, infertility, surgery, spontaneous pregnancy, *in vitro* fertilization

## Abstract

Endometriosis is a chronic disease that affects around 10% of reproductive age women worldwide and a common cause of infertility. One of its manifestations is ovarian endometriomas, which are present in 17-44% of endometriosis patients. Endometriomas can impair fertility by mechanical stretching and local inflammation, promoting oxidative stress in the surrounding ovarian cortex that could lead to apoptosis and necrosis of early follicles. The removal of endometriomas may improve spontaneous pregnancy rates, as already demonstrated by some studies. To reduce endometriomas recurrence, it is advised to perform cystectomy followed by hormonal suppression. However, this approach is unfeasible in patients desiring pregnancy. At the same time, cystectomy poses a threat to ovarian reserve and, therefore, to controlled ovarian stimulation. Women who have endometriomas surgically removed are at risk to have diminished response to ovarian stimulation if in vitro fertilization is needed in the future..

## INTRODUCTION

Endometriosis is a chronic disease characterized by the presence of endometrial tissue (glands and stroma) outside the uterine cavity. It is estimated to affect around 10% of women of reproductive age and is even more common in infertile patients (Supermaniam & Thye, 2021).

Endometriomas are the ovarian manifestation of the disease and are found in 17-44% of patients (Alborzi *et al.*, 2019). They are diagnosed by ultrasound scanning and are usually associated with advanced stages of endometriosis (Alborzi *et al.*, 2019). It is believed that endometriomas can affect ovarian physiology by mechanical stretching and local inflammation, promoting oxidative stress in the surrounding ovarian cortex that could lead to apoptosis and necrosis of early follicles (Leone Roberti Maggiore *et al.*, 2017).

Infertility is one of the challenges that women with endometriosis face and surgery is believed to increase pregnancy rates over no treatment or diagnostic laparoscopy (Bafort *et al.*, 2020; Hodgson *et al.*, 2020; Becker *et al.*, 2022). However, we believe that the surgical treatment for endometriomas, while improving the changes of natural pregnancy can, paradoxically, impair the outcomes of assisted reproductive treatment, if needed in the future.

### Endometrioma surgical treatment and spontaneous pregnancy

Endometriomas may have a negative impact on pregnancy rates and some studies have investigated whether their surgical removal is associated with higher changes of spontaneous pregnancy (Alborzi *et al.*, 2019; Leone Roberti Maggiore *et al.*, 2017; Maul *et al.*, 2014). There are no studies comparing the surgical removal of endometriomas with expectant management for this outcome.

Retrospective data on 550 women with ovarian endometriotic cysts who underwent cystectomy was analyzed. The follow up cohort was composed of 289 patients, of whom 111 women reported pregnancy desire. The postoperative spontaneous pregnancy rate was 54.1% (Maul *et al.*, 2014).

A cohort of 100 women who had surgery for endometrioma removal was followed for 12 months before being referred to assisted reproductive treatment (ART) if a spontaneous pregnancy was not achieved. Cystectomy was performed in 52 patients and fenestration and coagulation in 48 patients. The spontaneous pregnancy rate of infertile women was 59.4% in the cystectomy group and 23.3% in the fenestration group after one year of follow-up (Alborzi *et al.*, 2004). The same author performed a systematic review and meta-analysis of different endometrioma treatments for infertility, including surgery and assisted reproductive treatment. Although there were no statistically significant differences in clinical pregnancy rate between groups, the surgery-alone group had the highest pregnancy rate (43.8%) when compared to the other groups (surgery + ART, ART alone and sclerotherapy + ART) (Alborzi *et al.*, 2019).

A case series of 143 women who underwent endometrioma cystectomy for chronic pelvic pain and/or infertility evaluated pregnancy rate in the seventy-six patients who wished to become pregnant. The mean age of patients was 31.9 years. After surgery, 42.1% of women conceived spontaneously at a mean duration of 6.9 months and 6.6% of patients conceived twice after surgery (Supermaniam & Thye, 2021).

A study highlighted that ‘no RCT or meta-analysis are available to answer the question whether surgical excision of moderate to severe endometriosis enhances pregnancy rates’ (Vercellini *et al.*, 2009). The authors evaluated uncontrolled studies reporting the impact of laparoscopic treatment of endometriomas on reproductive outcomes. They found that the pregnancy rate varies from 30% to 67% in the studies, with an overall weighted mean of 50%. They acknowledge, however, the potential for selection and publication bias (Vercellini *et al.*, 2009). Another review on endometrioma treatment for fertility improvement found that cumulative pregnancy rates after surgery varied between 22.3% and 48.9%, with the highest rates being achieved after cystectomy technique (Leone Roberti Maggiore *et al.*, 2017).

The European Society for Human Reproduction and Embriology (ESHRE) acknowledges that data from comparative studies are lacking but clinicians may consider laparoscopic treatment of endometrioma in infertile patients for improving natural pregnancy chances (Becker *et al.*, 2022). As regarding the surgical technique, results seem to be best with cystectomy when compared to fenestration/coagulation and laser vaporization (Becker *et al.*, 2022).

## ENDOMETRIOMA SURGICAL TREATMENT AND RECURRENCE

Endometriosis surgery can relieve pain symptoms, restore pelvic anatomy, and improve fertility outcomes (Zakhari *et al.*, 2021; Koga *et al.*, 2015). Eradication of endometriotic lesions during surgery diminishes the chance of disease recurrence (Chiu *et al.*, 2022). However, reoperation rates are estimated to be between 27% and 58% when the ovaries are preserved (Zakhari *et al.*, 2021). Endometriomas could relapse due to regrowth of residual lesions, ovulation and retrograde menstruation leading to *de novo* lesions (Koga *et al.*, 2015; Chiu *et al.*, 2022). Most of recurrence cases involve the previously treated ovary (Koga *et al.*, 2015).

Surgery can remove all, or at least most, of the ectopic endometrial tissue. Therefore, post-operative hormonal suppression is a strategy that minimizes ovulation, endometrial cells activity and their risk of reimplanting in the peritoneal cavity (Zakhari *et al.*, 2021).

A systematic review and metanalysis evaluated women who underwent cystectomy for endometrioma followed by hormonal suppression or expectant management for at least 12 months. The authors found that hormonal treatment decreased endometrioma recurrence, with the best results for dienogest associated with gonadotropin releasing hormone agonist (GnRHa), followed by dienogest alone and oral combined contraceptive pill associated with GnRHa (Chiu *et al.*, 2022).

Koga et al. concluded that prevention of pain symptoms recurrence was only achieved with long-term oral contraceptive use (more than 6 months). Regarding endometrioma recurrence, oral contraceptive use for 2 years or more demonstrated significantly protective effect. Moreover, recurrence was frequently observed in patients who discontinued treatment. Regarding the administration regimen, the studies analyzed found no difference between cyclic or continuous regimens (Koga *et al.*, 2015).

A retrospective study reviewed data on 206 women surgically treated for moderate or severe endometriosis between 2004 and 2014. The stage of disease was not associated with recurrence risk. Interestingly, the recurrence rate was lower in patients who did not receive pharmacological treatment after surgery. The authors attribute this difference to the fact that most of these patients became pregnant after the procedure, which may have reduced the recurrence rate during the study time (Schippert *et al.*, 2020).

A systematic review and metanalysis concluded that patients receiving post-operative hormonal suppression therapy were significantly less likely to experience disease recurrence and had significantly lower pain scores when compared to controls (Zakhari *et al.*, 2021). On the other hand, a randomized controlled trial showed no difference in endometrioma recurrence after a year of LNG-IUS use compared to expectant management in patients undergoing laparoscopic cystectomy followed by 6 months of GnRHa (Chen *et al.*, 2017). This may suggest that ovulatory suppression after surgery is important to prevent ovarian disease recurrence.

Regarding surgical techniques, a Cochrane Systematic Review and Metanalysis concluded that excision of the cyst wall in endometriomas larger than 3 cm in size was associated with reduced recurrence rate of dysmenorrhea, dyspareunia, acyclic pelvic pain, and reduced recurrence of the endometrioma itself (Hart *et al.*, 2008).

Based on the above results and other several studies, ESHRE stated in the latest endometriosis guideline that “After surgical management of ovarian endometrioma in women not immediately seeking conception, clinicians are recommended to offer long-term hormone treatment for the secondary prevention of endometrioma and endometriosis-associated related symptom recurrence”. Regarding the type of procedure, cystectomy is probably superior to drainage and coagulation (Becker *et al.*, 2022).

Women who wish to become pregnant and have endometriomas could benefit from surgery to enhance cumulative pregnancy rates. Paradoxically, the best approach to prevent disease relapse cannot be offered to these patients. Therefore, while trying to conceive these women are subject to a greater risk of endometrioma recurrence.

## ENDOMETRIOSIS SURGERY, NATURAL PREGNANCY, AND ASSISTED REPRODUCTION

The choice to whether pursue a spontaneous pregnancy or directly initiate an assisted reproductive treatment after endometriosis surgery is a matter of debate. The endometriosis fertility index (EFI) is a staging system first published in 2010 to estimate the natural conception rate after laparoscopic surgery for endometriosis (Vesali *et al.*, 2020). It should be used for postoperative counselling to decide between assisted reproductive treatment and nonART management (Tomassetti, 2020).

The EFI considers historical factors, such as age, duration of infertility, and pregnancy history together with surgical factors. The latter are based on the AFS score and the so called ‘least function score’, determined by the surgeon at the end of the procedure for each of the tube, fimbria, and ovary. The sum of both historical and surgical factors results in the EFI score (Adamson & Pasta, 2010). The score ranges from 0 to 10 and estimates the natural pregnancy rate within 3 years after surgery. It has been externally validated since its first publication in 2010.

A systematic review and metanalysis of 17 studies that included over 3000 women found that the cumulative nonART pregnancy rate at 36 months was 10% for the lowest EFI score and 69% for the highest. The area under the curve (AUC) of the score for pregnancy prediction varied between 64% and 85%. The authors acknowledge, however, the high heterogeneity between studies (Vesali *et al.*, 2020). A retrospective cohort study of 235 patients with stage III/IV endometriosis who underwent fertility-sparing surgery showed that sixty-three percent of patients had a live birth following treatment, 64% of them without ART. The mean follow-up period was 4.1 years. The authors observed that women with an EFI score of 0-2 had no live births within 5 years of follow-up and this number steadily increased up to 91% with an EFI score of 9-10 (Maheux-Lacroix *et al.*, 2017).

The revised American Society for Reproductive Medicine (rASRM) score classifies endometriosis in minimal, mild, moderate, and severe disease (stages I, II, III and IV) (ASRM, 1997). According to this classification, ovarian endometrioma is already a marker of moderate disease. The presence of an endometrioma reduces the AFS score of the EFI to 0 and reduces the least function score (Adamson & Pasta, 2010). Therefore, even if the only endometriosis manifestation in the patient is a single endometrioma, and all the historical factors are of good prognosis, this patient will have an EFI of 8, which gives a cumulative pregnancy rate of 40% within one year after surgery (Adamson & Pasta, 2010).

According to the exposed above, at least 60% of patients with endometrioma will need assisted reproductive treatment after its removal. Surgery can also reduce ovarian reserve, as already demonstrated by the reduction of antimüllerian hormone levels (AMH) and antral follicle count (AFC) (Leone Roberti Maggiore *et al.*, 2017; Somigliana *et al.*, 2012). Cystectomy, despite being the best approach to increase spontaneous pregnancy rates and decrease recurrence rates, has a greater negative impact on AMH and AFC (Daniilidis *et al.*, 2023; Adamyan *et al.*, 2023). The damage to ovarian reserve is more pronounced in patients with bilateral endometriomas and cysts with a mean diameter >5cm (Daniilidis *et al.*, 2023). Therefore, it is reasonable to consider how much endometriomas could impact ovarian response to stimulation and if ovarian surgery would have an even more deleterious effect on IVF outcomes.

A study showed a reduced impact on ovarian reserve with less invasive methods, such as sclerotherapy, when compared to cystectomy. The sample size, however, was small (Vaduva *et al.*, 2023). A narrative review reported similar AMH decrement at 6 months postoperatively after sclerotherapy and cystectomy in two studies, but a significant negative impact on ovarian reserve after cystectomy in another study (Jee, 2022). Regarding pregnancy rates after IVF, data is still controversial, with some studies showing that the sclerotherapy group had better rates than the cystectomy group, while others show similar results. When analyzing spontaneous pregnancy rates after both treatments, the results are the same. It is also important to consider that, although a less invasive approach, sclerotherapy can have complications such as abdominal pain, ovarian abscess and intraperitoneal hemorrhage (Jee, 2022). Nevertheless, the procedure could be considered as an alternative management for endometriomas to minimize ovarian tissue damage in some cases.

The question of whether endometriomas affect ovarian response to controlled stimulation is limited by the fact that, in most cases, endometriomas are unilateral and therefore the contralateral ovary could compensate for the reduced function. Moreover, studies do not usually distinguish between patients who underwent endometrioma surgery before IVF and those who did not had surgery (Somigliana *et al.*, 2006). A systematic review and metanalysis of 17 studies reported fewer number of oocytes, mature oocytes and total embryos formed in patients with endometriomas compared to those without. When comparing only cases with unilateral endometriomas, the affected ovary had fewer co-dominant follicles than the intact one (Yang *et al.*, 2015).

A reduced responsiveness to gonadotropins in the presence of ovarian endometriomas was reported, which was more evident in women with larger cysts (Somigliana *et al.*, 2006). González-Foruria et al. retrospectively analyzed data on infertile patients with at least one ovarian endometrioma undergoing their first IVF/ICSI cycle compared with control women without endometriosis. They found endometrioma patients required significantly higher gonadotropin dose for stimulation and had a significantly lower number of follicles >14mm, oocytes retrieved and mature oocytes (González-Foruria *et al.*, 2020). Another study reported that the ovarian response to stimulation in terms of number of oocytes retrieved was poorer in endometrioma patients compared to tubal factor controls and decreased significantly in subsequent cycles (Al-Azemi *et al.*, 2000).

A multicenter retrospective cohort study tried to eliminate the bias of having a normal ovary compensating a contralateral endometrioma. The authors analyzed 39 women with unoperated bilateral endometriomas matched with 78 unexposed controls undergoing ovarian stimulation for IVF/ICSI and found fewer number of developing follicles and oocytes retrieved in the endometrioma patients’ group. The total dose of gonadotropins used and number of days of stimulation did not differ between cases and controls (Benaglia *et al.*, 2013).

Several studies suggest a reduced response to ovarian stimulation in women with endometriomas. It is also a matter of debate whether endometrioma surgery would impair or improve ovarian response and in vitro fertilization outcomes. Demirdag *et al.* compared the IVF outcomes of women previously operated for endometriomas with women who did not undergo surgery and a control group with no endometriosis. Their results showed that women with previous endometrioma surgery had fewer number of oocytes retrieved and mature oocytes, a higher cycle cancellation rate and a higher incidence of poor response to stimulation compared to the other groups. The clinical pregnancy rates and live birth rates, however, were similar between groups (Demirdag *et al.*, 2021).

A meta-analysis of 21 studies analyzed the pros and cons of stripping ovarian endometriomas before IVF/ICSI. The analysis showed that the total amount of gonadotropins used was higher in women who underwent endometrioma cystectomy, but the duration of stimulation was similar between groups. The number of dominant follicles and number of oocytes retrieved was lower in patients with previous surgery when compared to conservative approach. Despite the lower number of oocytes available, the total formed embryos, pregnancy and live birth rates were similar in the pooled analysis. The heterogeneity between studies was considered low (Tao *et al.*, 2017).

Endometrioma surgery is known to pose a threat to ovarian reserve (Tao *et al.*, 2017). Women with diminished ovarian reserve (DOR) may also have a poor response to ovarian stimulation (Conforti *et al.*, 2019). A retrospective case-control study tried to determine whether there were differences on IVF/ICSI outcomes between women with DOR following endometrioma surgical approach when compared to DOR without ovarian surgery. The authors found no differences between groups regarding number of oocytes retrieved and mature oocytes, cycle cancelation rates, total dose of gonadotropins administered and live birth rates (Hong *et al.*, 2017). These results indicate that the poorer outcomes of endometrioma surgery may be related to the impact on ovarian reserve rather than the disease itself.

Most studies that demonstrated a negative impact of endometrioma surgery on ovarian response to stimulation also showed no differences regarding pregnancy and live birth rates between groups (Al-Azemi *et al.*, 2000; Benaglia *et al.*, 2013; Demirdag *et al.*, 2021; Tao *et al.*, 2017). The obstetrical outcome in most studies is clinical pregnancy and live birth rate per embryo transfer (González-Foruria *et al*., 2020, Tao *et al*., 2017, Conforti *et al*., 2019) and not cumulative pregnancy rate per started cycle. Patients with previous endometrioma surgery have systematically a lower number of oocytes and embryos and, therefore, are expected to have a lower cumulative pregnancy rate and lower chances of a future pregnancy. Another point to consider is that the mean age of patients in those studies was less than 35 years old and in most of them, fertility duration was between 2 and 3 years (Al-Azemi *et al.*, 2000; Benaglia *et al.*, 2013; Tao *et al.*, 2017; Zeng *et al.*, 2022). Therefore, patients were considered of good prognosis. It is reasonable to consider that older women might have a greater deleterious impact of surgery on ovarian reserve and assisted reproductive outcomes. Indeed, Geber et al. stratified patients by age (<=35 years and >35years) and found that older patients with previous endometrioma surgery not only had less follicles and oocytes retrieved but also a lower pregnancy rate (34.5% *vs*. 48.3%). Although the latest outcome failed to reach statistical significance, their sample size was small (29 patients in each group) (Geber *et al.*, 2002).

Since maternal age is the most important factor determining the likelihood of conception in assisted reproductive treatments (Irani *et al.*, 2019), it is possible that in older patients the ART treatment outcomes after endometrioma surgery may be more severely affected. Also, in younger patients the aneuploidy rates are lower and, therefore, few oocytes are needed to obtain an euploid blastocyst (Haahr *et al.*, 2018). Considering that women are delaying childbirth, more patients will need assisted reproductive treatments in the future (Guzman *et al.*, 2019). For the ones affected by endometriosis, it is likely that ovarian surgery could be harmful for their reproductive future. Despite being the best approach for enhancing the chances of natural pregnancy, endometrioma cystectomy can paradoxically reduce the future pregnancy and cumulative live birth rates if IVF is needed, considering that older patients systematically have fewer oocytes and more aneuploid embryos.

## CONCLUSIONS

To enhance the spontaneous pregnancy rates in women with endometrioma, the surgical removal of cysts can be indicated in some cases. The gold standard technique is cystectomy and hormonal suppression is indicated to reduce recurrence rates. However, patients trying to conceive cannot be offered this approach and are subject to a greater disease recurrence rate. Moreover, endometrioma surgery can reduce ovarian reserve, which can be deleterious for assisted reproductive treatment, especially in women of advanced age. The paradox of the management of endometriomas in infertile patients is that approximately 60% of women with endometriomas will need ART in the future and, therefore, the procedure that can be offered to enhance spontaneous pregnancy rates in these patients can also worsen their future assisted reproductive outcomes.

## References

[r1] Adamson GD, Pasta DJ. (2010). Endometriosis fertility index: the new, validated endometriosis
staging system. Fertil Steril.

[r2] Adamyan L, Kasyan V, Pivazyan L, Isaeva S, Avetisyan J. (2023). Laser vaporization compared with other surgical techniques in
women with ovarian endometrioma: a systematic review and
meta-analysis. Arch Gynecol Obstet.

[r3] Al-Azemi M, Bernal AL, Steele J, Gramsbergen I, Barlow D, Kennedy S. (2000). Ovarian response to repeated controlled stimulation in in-vitro
fertilization cycles in patients with ovarian endometriosis. Hum Reprod.

[r4] Alborzi S, Momtahan M, Parsanezhad ME, Dehbashi S, Zolghadri J, Alborzi S. (2004). A prospective, randomized study comparing laparoscopic ovarian
cystectomy versus fenestration and coagulation in patients with
endometriomas. Fertil Steril.

[r5] Alborzi S, Zahiri Sorouri Z, Askari E, Poordast T, Chamanara K. (2019). The success of various endometrioma treatments in infertility: A
systematic review and meta-analysis of prospective studies. Reprod Med Biol.

[r6] Bafort C, Beebeejaun Y, Tomassetti C, Bosteels J, Duffy JM. (2020). Laparoscopic surgery for endometriosis. Cochrane Database Syst Rev.

[r7] Becker CM, Bokor A, Heikinheimo O, Horne A, Jansen F, Kiesel L, King K, Kvaskoff M, Nap A, Petersen K, Saridogan E, Tomassetti C, van Hanegem N, Vulliemoz N, Vermeulen N, ESHRE Endometriosis Guideline Group (2022). ESHRE guideline: endometriosis. Hum Reprod Open.

[r8] Benaglia L, Bermejo A, Somigliana E, Faulisi S, Ragni G, Fedele L, Garcia-Velasco JA. (2013). In vitro fertilization outcome in women with unoperated bilateral
endometriomas. Fertil Steril.

[r9] Chen YJ, Hsu TF, Huang BS, Tsai HW, Chang YH, Wang PH. (2017). Postoperative maintenance levonorgestrel-releasing intrauterine
system and endometrioma recurrence: a randomized controlled
study. Am J Obstet Gynecol.

[r10] Chiu CC, Hsu TF, Jiang LY, Chan IS, Shih YC, Chang YH, Wang PH, Chen YJ. (2022). Maintenance Therapy for Preventing Endometrioma Recurrence after
Endometriosis Resection Surgery - A Systematic Review and Network
Meta-analysis. J Minim Invasive Gynecol.

[r11] Conforti A, Esteves SC, Picarelli S, Iorio G, Rania E, Zullo F, De Placido G, Alviggi C. (2019). Novel approaches for diagnosis and management of low prognosis
patients in assisted reproductive technology: the POSEIDON
concept. Panminerva Med.

[r12] Daniilidis A, Grigoriadis G, Kalaitzopoulos DR, Angioni S, Kalkan Ü, Crestani A, Merlot B, Roman H. (2023). Surgical Management of Ovarian Endometrioma: Impact on Ovarian
Reserve Parameters and Reproductive Outcomes. J Clin Med.

[r13] Demirdag E, Guler I, Selvi I, Cevher Akdulum MF, Canan S, Erdem A, Erdem M. (2021). Analysis of 2438 cycles for the impact of endometrioma and its
surgery on the IVF outcomes. Eur J Obstet Gynecol Reprod Biol.

[r14] Geber S, Ferreira DP, Spyer Prates LF, Sales L, Sampaio M. (2002). Effects of previous ovarian surgery for endometriosis on the
outcome of assisted reproduction treatment. Reprod Biomed Online.

[r15] González-Foruria I, Soldevila PB, Rodríguez I, Rodríguez-Purata J, Pardos C, García S, Pascual MÁ, Barri PN, Polyzos NP. (2020). Do ovarian endometriomas affect ovarian response to ovarian
stimulation for IVF/ICSI?. Reprod Biomed Online.

[r16] Guzman L, Inoue N, Núñez D, Meza J, Bendezu P, Pino P, Portella J, Noriega-Portella L, Noriega-Hoces L. (2019). What advice should we give our patients to preserve their
fertility and avoid needing oocyte donation in the future? - A Social
Fertility Preservation program. JBRA Assist Reprod.

[r17] Haahr T, Esteves SC, Humaidan P (2018). Individualized controlled ovarian stimulation in expected
poor-responders: an update. Reprod Biol Endocrinol.

[r18] Hart RJ, Hickey M, Maouris P, Buckett W. (2008). Excisional surgery versus ablative surgery for ovarian
endometriomata. Cochrane Database Syst Rev.

[r19] Hodgson RM, Lee HL, Wang R, Mol BW, Johnson N. (2020). Interventions for endometriosis-related infertility: a systematic
review and network meta-analysis. Fertil Steril.

[r20] Hong SB, Lee NR, Kim SK, Kim H, Jee BC, Suh CS, Kim SH, Choi YM. (2017). In vitro fertilization outcomes in women with surgery induced
diminished ovarian reserve after endometrioma operation: Comparison with
diminished ovarian reserve without ovarian surgery. Obstet Gynecol Sci.

[r21] Irani M, Zaninovic N, Rosenwaks Z, Xu K. (2019). Does maternal age at retrieval influence the implantation
potential of euploid blastocysts?. Am J Obstet Gynecol.

[r22] Jee BC. (2022). Efficacy of ablation and sclerotherapy for the management of
ovarian endometrioma: A narrative review. Clin Exp Reprod Med.

[r23] Koga K, Takamura M, Fujii T, Osuga Y. (2015). Prevention of the recurrence of symptom and lesions after
conservative surgery for endometriosis. Fertil Steril.

[r24] Leone Roberti Maggiore U, Gupta JK, Ferrero S. (2017). Treatment of endometrioma for improving fertility. Eur J Obstet Gynecol Reprod Biol.

[r25] Maheux-Lacroix S, Nesbitt-Hawes E, Deans R, Won H, Budden A, Adamson D, Abbott JA. (2017). Endometriosis fertility index predicts live births following
surgical resection of moderate and severe endometriosis. Hum Reprod.

[r26] Maul LV, Morrision JE, Schollmeyer T, Alkatout I, Mettler L. (2014). Surgical therapy of ovarian endometrioma: recurrence and
pregnancy rates. JSLS.

[r27] (1997). Revised American Society for Reproductive Medicine classification
of endometriosis: 1996. Fertil Steril.

[r28] Schippert C, Witte Y, Bartels J, Garcia-Rocha GJ, Jentschke M, Hillemanns P, Kundu S. (2020). Reproductive capacity and recurrence of disease after surgery for
moderate and severe endometriosis - a retrospective single center
analysis. BMC Womens Health.

[r29] Somigliana E, Berlanda N, Benaglia L, Viganò P, Vercellini P, Fedele L. (2012). Surgical excision of endometriomas and ovarian reserve: a
systematic review on serum antimüllerian hormone level
modifications. Fertil Steril.

[r30] Somigliana E, Infantino M, Benedetti F, Arnoldi M, Calanna G, Ragni G. (2006). The presence of ovarian endometriomas is associated with a
reduced responsiveness to gonadotropins. Fertil Steril.

[r31] Supermaniam S, Thye WL. (2021). Laparoscopic cystectomy in treating women with endometrioma and
pregnancy outcome - a case series. Med J Malaysia.

[r32] Tao X, Chen L, Ge S, Cai L. (2017). Weigh the pros and cons to ovarian reserve before stripping
ovarian endometriomas prior to IVF/ICSI: A meta-analysis. PLoS One.

[r33] Tomassetti C. (2020). Why and when you should use the endometriosis fertility index
(EFI). BJOG.

[r34] Vaduva CC, Dira L, Carp-Veliscu A, Goganau AM, Ofiteru AM, Siminel MA. (2023). Ovarian reserve after treatment of ovarian endometriomas by
ethanolic sclerotherapy compared to surgical treatment. Eur Rev Med Pharmacol Sci.

[r35] Vercellini P, Somigliana E, Viganò P, Abbiati A, Barbara G, Crosignani PG. (2009). Surgery for endometriosis-associated infertility: a pragmatic
approach. Hum Reprod.

[r36] Vesali S, Razavi M, Rezaeinejad M, Maleki-Hajiagha A, Maroufizadeh S, Sepidarkish M. (2020). Endometriosis fertility index for predicting non-assisted
reproductive technology pregnancy after endometriosis surgery: a systematic
review and meta-analysis. BJOG.

[r37] Yang C, Geng Y, Li Y, Chen C, Gao Y. (2015). Impact of ovarian endometrioma on ovarian responsiveness and IVF:
a systematic review and meta-analysis. Reprod Biomed Online.

[r38] Zakhari A, Delpero E, McKeown S, Tomlinson G, Bougie O, Murji A. (2021). Endometriosis recurrence following post-operative hormonal
suppression: a systematic review and meta-analysis. Hum Reprod Update.

[r39] Zeng C, Lu R, Li X, Kuai Y, Wang S, Xue Q. (2022). The presence of ovarian endometrioma adversely affect ovarian
reserve and response to stimulation but not oocyte quality or IVF/ICSI
outcomes: a retrospective cohort study. J Ovarian Res.

